# The *SOX2 *response program in glioblastoma multiforme: an integrated ChIP-seq, expression microarray, and microRNA analysis

**DOI:** 10.1186/1471-2164-12-11

**Published:** 2011-01-06

**Authors:** Xuefeng Fang, Jae-Geun Yoon, Lisha Li, Wei Yu, Jiaofang Shao, Dasong Hua, Shu Zheng, Leroy Hood, David R Goodlett, Gregory Foltz, Biaoyang Lin

**Affiliations:** 1 Swedish Neuroscience Institute, Swedish Medical Center, Seattle, WA 98122, USA; 2 Zhejiang-California International NanoSystems Institute, Zhejiang University, Hangzhou, Zhejiang 310029, PR China; 3 Cancer Institute (Key Laboratory of Cancer Prevention and Intervention, China National Ministry of Education), The Second Affiliated Hospital, Zhejiang University School of Medicine, Hangzhou, Zhejiang 310009, PR China; 4 Department of Urology, University of Washington, Seattle, WA 98195, USA; 5 Department of Medicinal Chemistry, University of Washington, BOX 367610, Seattle, WA 98195, USA; 6 The Institute for Systems Biology, Seattle, WA 98103, USA

## Abstract

**Background:**

*SOX2 *is a key gene implicated in maintaining the stemness of embryonic and adult stem cells. *SOX2 *appears to re-activate in several human cancers including glioblastoma multiforme (GBM), however, the detailed response program of *SOX2 *in GBM has not yet been defined.

**Results:**

We show that knockdown of the *SOX2 *gene in LN229 GBM cells reduces cell proliferation and colony formation. We then comprehensively characterize the *SOX2 *response program by an integrated analysis using several advanced genomic technologies including ChIP-seq, microarray profiling, and microRNA sequencing. Using ChIP-seq technology, we identified 4883 *SOX2 *binding regions in the GBM cancer genome. *SOX2 *binding regions contain the consensus sequence wwTGnwTw that occurred 3931 instances in 2312 *SOX2 *binding regions. Microarray analysis identified 489 genes whose expression altered in response to *SOX2 *knockdown. Interesting findings include that *SOX2 *regulates the expression of SOX family proteins *SOX1 *and *SOX18*, and that *SOX2 *down regulates *BEX1 *(brain expressed X-linked 1) and *BEX2 *(brain expressed X-linked 2), two genes with tumor suppressor activity in GBM. Using next generation sequencing, we identified 105 precursor microRNAs (corresponding to 95 mature miRNAs) regulated by *SOX2*, including down regulation of miR-143, -145, -253-5p and miR-452. We also show that miR-145 and *SOX2 *form a double negative feedback loop in GBM cells, potentially creating a bistable system in GBM cells.

**Conclusions:**

We present an integrated dataset of ChIP-seq, expression microarrays and microRNA sequencing representing the *SOX2 *response program in LN229 GBM cells. The insights gained from our integrated analysis further our understanding of the potential actions of *SOX2 *in carcinogenesis and serves as a useful resource for the research community.

## Background

The SOX (SRY-like HMG box) gene family represents a family of transcriptional factors characterized by the presence of a homologous sequence called the HMG (high mobility group) box. The HMG box is a DNA binding domain that is highly conserved throughout eukaryotic species. So far, twenty SOX genes have been identified in humans and mice and they can be divided into 10 subgroups on the basis of sequence similarity and genomic organization [1,2]. SOX genes bind to the minor groove in DNA to control diverse developmental processes [3].

*SOX2*, one of the key members of the *SOX *family gene, is highly expressed in embryonic stem cells [4]. Recently, Takahashi et al. showed that *SOX2 *is a key transcription factor, in conjunction with *KLF4*, *OCT4 *and *c-Myc*, whose over expression can induce pluripotency in both mice and human somatic cells [5,6]. *SOX2 *is one of the four factors (*OCT4, SOX2, NANOG, and LIN28*) that Yu et al. used to reprogram human somatic cells to pluripotent stem cells that exhibit the essential characteristics of embryonic stem (ES) cells [7]. *SOX2 *is one of the two factors (*SOX2 *and *OCT4*) that were sufficient to generate induced pluripotent stem cells from human cord blood cells [8]. Due to its importance in conferring stemness of cells, the target genes for *SOX2 *in mouse embryonic stem cells were defined using ChIP-seq technology [9].

*SOX2 *has also been implicated in several cancers including gastric cancer [10,11], breast cancer [12,13], pancreatic cancer [14], pulmonary non-small cell and neuroendocrine carcinomas [15]. In addition, *SOX2 *was identified to be a prognostic marker for human esophageal squamous cell carcinoma [16] and rectal cancer [17]. Schmitz *et al*. found that *SOX2 *is over expressed in malignant glioma while displaying minimal expression in normal tissues [18]. More recently, Gangemi *et al*. showed that silencing of the *SOX2 *in freshly derived glioblastoma tumor-initiating cells (TICs) stopped proliferation and the resulting cells lost tumorigenicity in immunodeficient mice [19]. Ikushima *et al*. showed that inhibition of TGF-beta signaling drastically deprived tumorigenicity of glioma-initiating cells (GICs) by promoting their differentiation, and that these effects were attenuated in GICs transduced with *SOX2 *or *SOX4 *[20]. Taking together, these data suggested that *SOX2 *is also a key gene in maintaining the stemness of glioma stem cells.

Given that *SOX2 *is predominantly expressed in embryonic and adult stems cells, including neural progenitor cells, and re-activates in cancers, including malignant gliomas, we hypothesized that the re-activation program of *SOX2 *may play an important role in the carcinogenesis and maintenance of GBM. Although the *SOX2 *response program in mouse stem cells was previously defined [9], the re-activation program in cancers such as GBM has not yet been defined. Using ChIP-seq technology, we conducted a genome-wide target identification for *SOX2 *binding in GBM cells. We generated mRNA expression profiles using the Applied Biosystems' microarray platform and microRNA expression profiles using next-generation sequencing after knockdown of *SOX2 *expression in GBM cells. An integrated analysis of these data reveals key response programs that potentially play important roles in GBM.

## Results

### *SOX2 *affects colony formation and cell proliferation in GBM

We previously completed massively parallel signature sequencing (MPSS) and identified *SOX2 *as significantly over expressed in GBM tissues compared to normal brain tissues [21]. We identified two MPSS tags that correspond to different polyadenylated isoform, and both are up-regulated in GBM tissues compared to normal brain tissues [21]. Our data is consistent with the observation that *SOX2 *is widely expressed in gliomas including glioblastomas but not in normal brains except for in ependymal layers [22].

**Figure 1:**
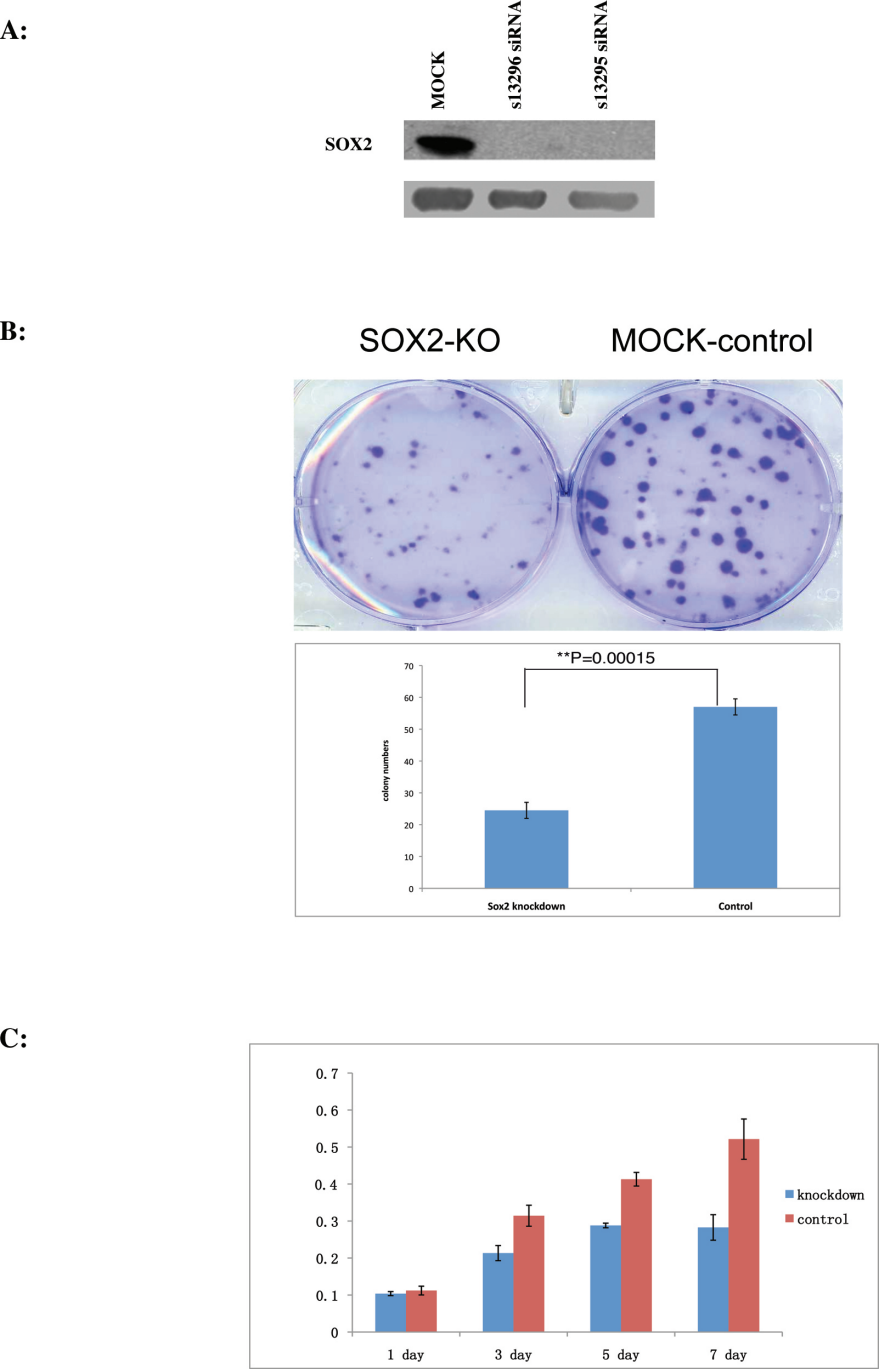
**Functional analysis of *SOX2 *in GBM cells**. (A) Western blot analysis showing the known down efficiency of *SOX2 *siRNAs (s13295 and s13296). (B) Colony formation assays for *SOX2 *knockdown and mock control. (C). Cell proliferation assays for *SOX2 *knockdown and mock control.

To assess the functional consequences of *SOX2*, we knocked down the *SOX2 *gene by siRNAs in the GBM cell line LN229 using *SOX2 *SiRNAs (Ambion Inc.). As shown in Figure 1A, we were able to knockdown *SOX2 *almost completely using either of the pre-designed *SOX2 *siRNAs (s13295 and s13296) from Ambion Inc. (Now Applied Biosystems Inc.).

Knockdown of the *SOX2 *gene in LN229 cells significantly reduced the numbers of colonies formed as shown in Figure 1B. In three replicate experiments, the colony numbers for the MOCK-knockdown cells were 53.3 (STDEV = 2.5) while that for the *SOX2 *knockdown were 24.7 (STDEV = 2.5) (T-test P = 0.00015, 2 tails, type 2). Furthermore, knockdown of *SOX2 *in LN229 cells reduced the numbers of cells, reaching statistical significance at day four (T-test P < 0.001) and further at day six (T-test P = 1.45E-06) by MTT assays (Figure 1C).

### Global identification of *SOX2 *binding sites in GBM cells by ChIP-seq analysis

In order to understand the genome-wide binding patterns of *SOX2*, we applied ChIP-seq technology, which is a novel approach for identifying transcription factor binding sites genome-wide [23,24]. We performed replicate *SOX2 *ChIP and IgG ChIP. After sequencing analysis, we obtained a total of 1,139,535 and 638,279 sequence tags respectively for *SOX2 *and IgG that can be mapped uniquely to the human genome allowing two mismatches.

**Figure 2:**
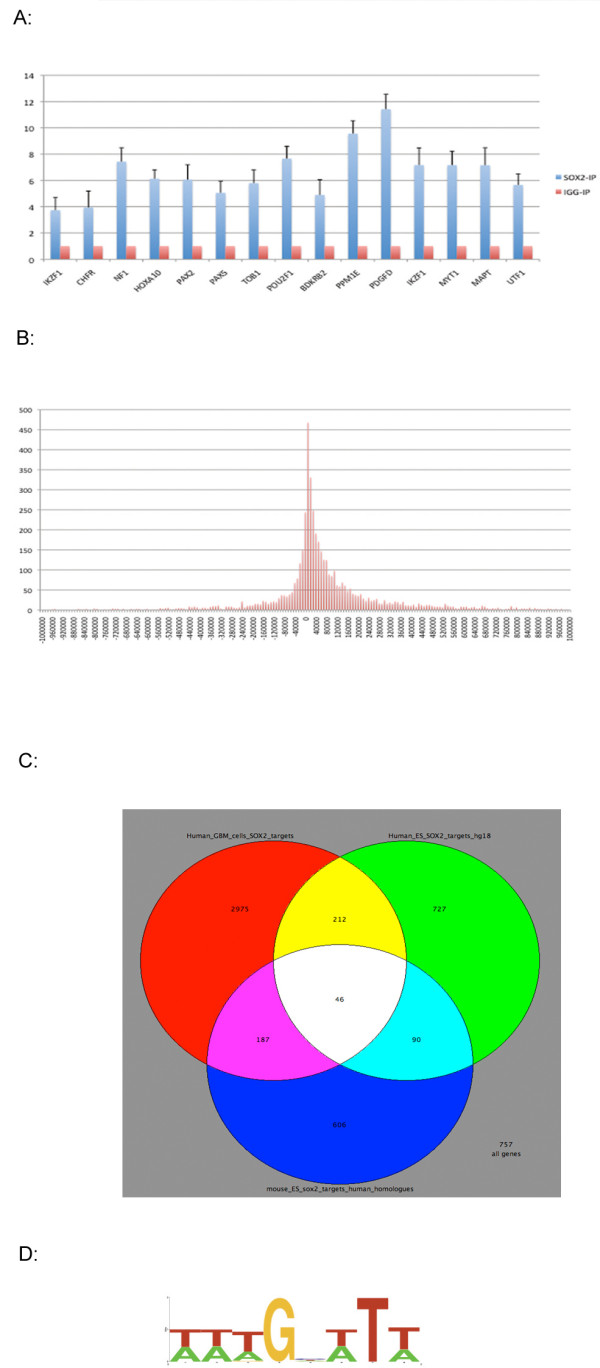
**ChIP-seq analysis of *SOX2 *in GBM cells**. Quantitative real time PCR for the confirmation of ChIP-seq peaks. Relative amount of PCR products from *SOX2*-ChIP and IgG-ChIP were shown as bar graph with the amount of IgG-ChIP normalized to 1. Standard deviations were also shown for *SOX2*-IP. (B) Histograms of *SOX2 *binding sites around annotated TSS (Transcription start sites) Frequencies of *SOX2 *island binding were calculated every 10 kilobases (Y-axis). Relative distance to TSS is shown in X-axis, Negative and positive values indicate localization 5' or 3' to TSS respectively. (C). A VENN diagram showing the overlaps of *SOX2 *targets among human GBM cells, human ES cells and mouse ES cells. (D). The consensus sequence wwTGnwTw with log-likelihood score of 13920.71 identified by the MotifSampler program. The over represented sequences were used as input for the Weblog program (http://weblogo.berkeley.edu/) [53] to display the consensus sequence graphically.

Using the SISSRs (Site Identification from Short Sequence Reads) ChIP-seq analysis program [25], we identified a total of 4,883 *SOX2 *binding regions with a P value < 0.01 using IgG control ChIP-seq data as the negative control (Additional File 1). We randomly picked 15 genes for which the promoter regions are enriched for the *SOX2 *IP, and we were able to confirm all 15 genes to be enriched in the *SOX2 *IP DNAs compared to the IgG-IP DNAs using real time quantitative PCR (Figure 2A), suggesting that the false positive rate is negligible in our dataset.

There are 4714 *SOX2 *binding regions that can be mapped to TSS (transcription start site) of 3420 known genes. We calculated the distance of the *SOX2 *binding regions to TSS (transcription start sites) and then tabulated the frequency across the distance intervals before TSS and after TSS. Figure 2B shows that the peak of the *SOX2 *binding regions is around the TSS sites. We found that about 13% *SOX2 *(605 of 4714) binding regions are mapped within 8 kb of TSS (Figure 2B), and about 25% (1161 binding regions) are mapped > 8 kb 5' distal to the TSS. The rest mapped to > 8 kb downstream of TSS start sites of genes.

To understand the function of the *SOX2 *binding genes, we performed a GO analysis using the GO miner program using evidence level 3 of molecular functions. The enriched GO terms are shown in Table 1. The top enriched GO terms include GO:0005096 GTPase activator activity, GO:0022843 voltage-gated cation channel activity, GO:0008066 glutamate receptor activity, GO:0005070 SH3 SH2 adaptor activity, GO:0005001 transmembrane receptor protein tyrosine phosphatase activity (Table 1). It should be pointed that expression of 196 of 792 genes with signal transducer activity, (GO:0004871), 137 of 562 receptor genes (GO:0004872) (among them, 101 of 410 transmembrane receptor genes, GO: 0004888), and 92 of 365 kinase genes (GO:0016301) (Table 1) was altered in response to *SOX2 *knockdown.

**Table 1:** GO terms that are enriched in SOX2 binding genes identified by ChIP-seq

GO CATEGORY	TOTAL GENES	Changed Genes	Enrichment folds	P value (Log10)	FDR
GO:0005096_GTPase_activator_activity	60	27	2.39	-5.54	0.01
GO:0004871_signal_transducer_activity	792	196	1.31	-5.17	0.003333
GO:0060089_molecular_transducer_activity	792	196	1.31	-5.17	0.003333
GO:0060090_molecular_adaptor_activity	48	20	2.21	-3.69	0.0175
GO:0022843_voltage-gated_cation_channel_activity	66	25	2.01	-3.67	0.014
GO:0008066_glutamate_receptor_activity	20	11	2.92	-3.50	0.016667
GO:0004872_receptor_activity	562	137	1.29	-3.41	0.02
GO:0005070_SH3_SH2_adaptor_activity	43	18	2.22	-3.40	0.02
GO:0004672_protein_kinase_activity	278	75	1.43	-3.37	0.017778
GO:0005001_transmembrane_receptor_protein_tyrosine_phosphatase_activity	18	10	2.95	-3.27	0.019091
GO:0019198_transmembrane_receptor_protein_phosphatase_activity	18	10	2.95	-3.27	0.019091
GO:0005099_Ras_GTPase_activator_activity	28	13	2.47	-3.11	0.023333
GO:0030695_GTPase_regulator_activity	124	38	1.63	-3.02	0.023077
GO:0016301_kinase_activity	365	92	1.34	-2.92	0.027143
GO:0042805_actinin_binding	4	4			
GO:0008092_cytoskeletal_protein_binding	164	47	1.52	-2.89	0.026667
GO:0016773_phosphotransferase_activity__alcohol_group_as_acceptor	326	83	1.35	-2.82	0.029375
GO:0004888_transmembrane_receptor_activity	410	101	1.31	-2.80	0.029412
GO:0005244_voltage-gated_ion_channel_activity	76	25	1.75	-2.63	0.035263
GO:0022832_voltage-gated_channel_activity	76	25	1.75	-2.63	0.035263
GO:0030674_protein_binding__bridging	58	20	1.83	-2.47	0.0455

Marson *et al*. and Chen *et al*. recently used ChIP-seq to map binding sites of *SOX2 *and other key TFs in the mouse ES cells [9,26]. Morsen *et al*. identified 4,087 *SOX2 *binding sites corresponding to 2,884 genes based on the criteria that a binding site is within 50 kb of the TSS or TES (transcript end site). Chen *et al*. identified 4,526 *SOX2 *binding regions (from their Supplementary Table 3) that could be assigned to 2,601 genes of the gene using the same criteria. The union of the two lists generated 4,380 genes. Interestingly, the overlapped genes between the two lists is 1105 genes (25.2%) (Additional File 2). The difference could be due to the use of different antibodies, Chen *et al*. used the *SOX2 *antibody (sc-17320, Santa Cruz Inc) while Morson *et al*. used an affinity purified goat polyclonal antibody (AF2018, R&D Systems), or differences in the analysis pipeline and down stream analysis procedures [9,26].

Using the homologene table for human and mouse from NCBI (http://www.ncbi.nlm.nih.gov/homologene), we compared the *SOX2 *targets that we identified in LN229 cells with the *SOX2 *targets that were identified in mouse ES cells [26]. We were able to identify 929 human homologues of 1105 mouse *SOX2 *binding genes from Chen *et al*'s paper, and then were able to identify 233 unique genes (25%) (Additional File 1) that are common to the *SOX2 *binding gene in the human GBM cells (Figure. 2C). These suggest that there are common sets of genes regulated by *SOX2 *in humans and mice, and in ES cells and in cancer cells. However, we identified many *SOX2 *binding sites that are only present in the glioblastoma cell line, suggesting that *SOX2 *targets different pathways in the context of cancer cells.

Boyer *et al*. applied ChIP-chip technology to identify *OCT4, SOX2*, and *NANOG *target genes in human embryonic stem cells using a human promoter array [27]. They identified 1,271 of the *SOX2 *binding promoter regions for known protein-coding genes in human ES cells. In LN229 cells, we found 258 unique genes that overlapped with their data (Additional File 1 and Figure. 2C). Analysis with the Fisher's Exact test (one sided) revealed that the overlap is highly significant (P < 0.001). This suggests that while there is some conservation of the genes regulated by *SOX2 *in ES cells and GBM cells, there are also differences in *SOX2 *binding regions between the cells. The difference could be due to several factors. First, there are differences in technologies used. The array designed by Boyer *et al*. covered the -8 kb to +2 kb region relative to each transcription start site of 18,002 transcription start sites representing 17,917 unique genes. If a *SOX2 *binding region is outside of the region covered by the printed oligos, or is not on the array, it would be missed by ChIP-chip analysis. However, the ChIP-seq technology is not limited by the probes selected to be printed on a chip, and therefore could identify *SOX *binding regions further upstream or down stream of genes. Second, the *SOX2 *response program could be different in different cells (i.e. GBM vs. ES cells). It is possible that different SOX proteins interact selectively with and regulate a unique repertoire of target genes, and the selectivity is dependent on the type of cell in which the protein is expressed.

To see whether they were unique functional classification or over representation for the *SOX2 *targets in GBM cells versus those in human ES cells, we compared 3162 unique *SOX2 *targets in GBM cells with 817 unique *SOX2 *targets in human ES cells using GSEA to identify unique over represented GO terms in each set of targets. We found that the unique *SOX2 *targets in GBM cells were enriched for ion transport, receptor activities, neuron differentiation neurogenesis, etc. (Additional File 3), while the unique *SOX2 *targets in human ES cells are enriched for macromolecular complex, ion homeostasis, apoptotic program, ATPase activity, etc. (Additional File 4).

We were interested to see whether the genes related to stemness and/or differentiation are *SOX2 *targets in GBM cells. Using the molecular signature database (http://www.broadinstitute.org/gsea/msigdb/index.jsp) at the Broad Institute, we found that GO term GO:0045595 (REGULATION OF CELL DIFFERENTIATION) consists of a compiled set of 59 genes related to differentiation. In addition, Ben-Porath *et al*. curated a gene set with 378 genes over expressed in human embryonic stem cells according to 5 or more out of 20 profiling studies in the Table S1 of their published paper [28]. We found that 17 of 59 genes related to regulation of cell differentiation were *SOX2 *targets in GBM cells including *ACIN1 *(apoptotic chromatin condensation inducer 1), *BMPR1B *(bone morphogenetic protein receptor, type IB), *ETS1 *(V-ets erythroblastosis virus E26 oncogene homolog 1), *SHH *(sonic hedgehog homolog, *Drosophila*), *IGFBP3 *(insulin-like growth factor binding protein 3) and *RUNX1 *(Runt-related transcription factor 1) (Additional File 5). In addition, 71 of 378 ES enriched genes were *SOX2 *targets in GBM cells including *CDC20 *(cell division cycle 20 homolog of *S. cerevisiae*), *CHEK2 *(CHK2 checkpoint homolog of *S. pombe*), *FGF13 *(fibroblast growth factor 13), *RFC3 *[replication factor C (activator 1) 3, 38 kDa] and *UTF1 *(undifferentiated embryonic cell transcription factor 1) (Additional File 6). However, we did not find *OCT4 *and *NANOG *to be *SOX2 *targets in GBM cells.

### Identification of the DNA binding consensus and other known TF binding sites in the *SOX2 *bound regions

To see whether the human *SOX2 *binding regions in GBM cells have their own unique and enriched binding motif, we used the MotifSampler program (http://bioinformatics.psb.ugent.be/webtools/plantcare/html/Motif_Sampler.html) to identify binding consensus sequences enriched in the *SOX2 *binding regions that we identified. We found a consensus sequence wwTGnwTw with a very high log-likelihood score of 13920.71. The output matrix for this consensus sequence is shown in Additional File 7, and there are 3931 instances of this motif in 2312 *SOX2 *binding regions (Additional File 8). The consensus logo is shown in Figure 2D.

We were curious whether known TFs could bind to the *SOX2 *binding regions that we identified and act as *SOX2 *cooperators for the regulation of gene expression. In order to systematically search for potential bindings of other transcription factors, we used the MotifScanner program (http://bioinformatics.psb.ugent.be/webtools/plantcare/html/Motif_Sampler.html) and scanned all TF motif matrices (PWM databases) using the human transcription factor subset of the Transfac professional 7.0. Matched matrices with likelihood (LR) ratios of 500 or higher were tabulated and frequencies calculated (Additional File 9). Among the top known TF matrices (Table 2) that were identified as co-occurred more than 10% of the time with *SOX2 *in the *SOX2*-binding region are: the OCT family, the FOX family, the HNF family, the GATA family and several other TF *IRF1 *(interferon regulatory factor 1), *POU1F1 *(POU class 1 homeobox 1), *TEF1 *(*TEAD1*, *TEA *domain family member 1), *AREB6 *(*ZEB1*, zinc finger E-box binding homeobox 1) and *GR *(glucocorticoid receptor, also named NR3C1, nuclear receptor subfamily 3, group C, member 1).

**Table 2:** Top known TFs binding sites in the SOX2 binding regions

Known TFs	Number of occurrencies	Percentage of total sites
AREB6_04	543	11.12
FOX_Q2	596	12.21
FOXD3_01	654	13.39
FOXJ2_01	506	10.36
FOXO1_01	519	10.63
GATA_C	524	10.73
GATA_Q6	647	13.25
GATA1_04	497	10.18
GR_Q6_01	491	10.06
HFH3_01	525	10.75
HNF1_Q6	530	10.85
HNF3_Q6	528	10.81
HNF3ALPHA_Q6	649	13.29
IRF1_Q6	702	14.38
OCT_Q6	594	12.16
OCT1_04	697	14.27
OCT1_06	554	11.35
OCT1_Q5_01	616	12.62
PIT1_Q6	571	11.69
POU1F1_Q6	633	12.96
TEF1_Q6	776	15.89

*POU1F1 *is the POU class 1 homeobox 1. OCT family TFs also contain POU domains. These suggest that *SOX2 *and many POU domain proteins may act together to control gene expression. *SOX2 *and OCT family TFs such as *OCT1 *(POU2F1, POU class 2 homeobox 1) and *OCT3/4 *(*POU5F1*, *POU *class 5 homeobox 1) are well known to work synergistically in embryonic stem cells [29,30]. Additionally, we identified novel transcriptional factors in the *SOX2 *bound regions, including FOX (fork head transcription factor) and HNF (hepatocyte nuclear factor) family proteins. However, its significance remains to be determined. Interestingly, *HNF1 *(hepatocyte nuclear factor 1) also contains a POU-homeodomain, while *HNF3 *alpha, which is also named *FOXA1*, contains a fork head domain [31].

### Microarray analysis reveals that *SOX2 *knockdown reduces the expression of other *SOX *family members but up-regulates *BEX1 *and *BEX2*

**Figure 3:**
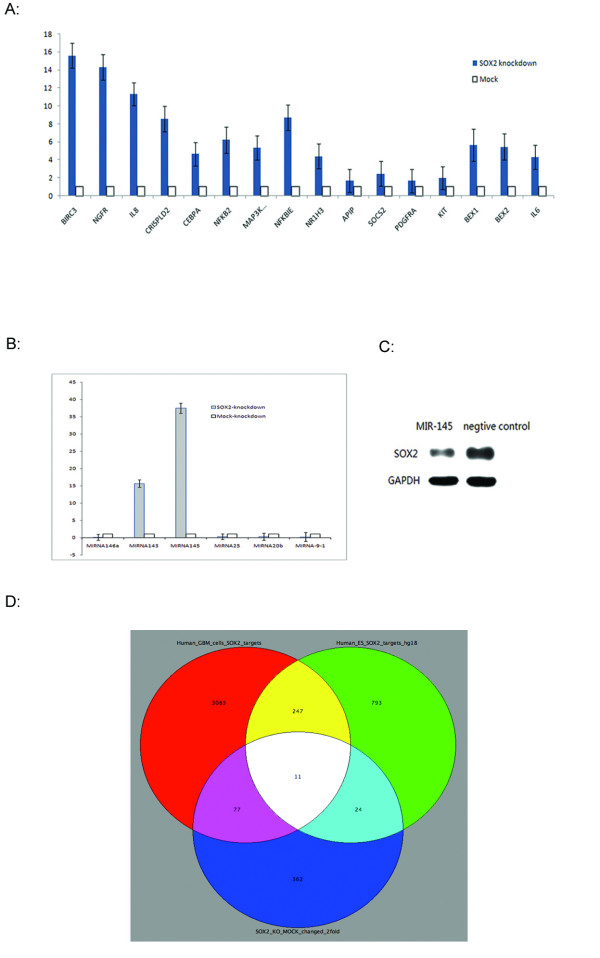
**Confirmation of *SOX2 *regulated genes and microRNAs**. (A) Bar graph showing PCR confirmation of the array data of *SOX2 *gene expression. *SOX2*-KO: *SOX2 *knockdown; MOCK, mock known down with negative control siRNAs. Y-axis, relative expression after normalizing the MOCK control to value of 1; X-axis, gene symbols. (B). Bar graph showing PCR confirmation of the microRNA sequencing data. Y-axis, relative expression after normalizing the MOCK control to value of 1; X-axis, microRNA names. (C). Western blot analysis showing the *SOX2 *expression comparing microRNA 145 precursor mimics and a scrambled negative control. (D). A VENN diagram showing the overlaps of *SOX2 *targets in GBM and ES cells, and the genes that changed expression (2 fold cutoff) after *SOX2 *knockdown in GBM cells.

We performed microarray analysis comparing *SOX2 *knockdown and MOCK transfected LN229 cells, and we identified a total of 565 probes (489 known genes with annotations) that were changed > 2 fold between *SOX2 *knockdown and *SOX2*-MOCK transfected LN229 cells (Additional File 10). Array analysis confirmed that *SOX2 *expression was indeed decreased after *SOX2 *knockdown. Additionally, we confirmed the array data by RT-PCR analysis of 13 randomly selected up-regulated genes after *SOX2 *knockdown (*BIRC3, NGFR, IL8, CRISPD2, CEBPA, NFKB2, MAP3K14, NFKBIE, NR1H3, APIP, SOCS2, PDGFRA, KIT, BEX1, BEX2, IL-6*) (Figure. 3A).

Gene Ontology analysis of all GO categories revealed that proteins belonging to these cellular locations GO:0005576 extracellular region and GO:0005796 Golgi lumen are enriched, and that proteins involved in the GO:0042035 regulation of cytokine biosynthetic process (Table 3). Indeed, we found that knockdown of *SOX2 *increased the expression of several cytokines including *IL6 *and *IL8, IL23, IL24 *and *IL32 *(Table 4), and the expression of two interleukin receptors-interleukin 7 receptor and interleukin 1 receptor-like 1. We also found many interesting families of proteins that were regulated by *SOX2 *by visual inspection of the gene list. For example, we noticed that two other SOX family protein, *SOX1 *and *SOX18*, also exhibited reduced expression by more than 3 fold after *SOX2 *knockdown (Table 4). We also found that many members of the protocadherins including protocadherin 9, 10, beta 11, and gamma A3 and C3 were reduced after *SOX2 *knockdown. However, the expression of protocadherin alpha 4 was increased after *SOX2 *knockdown (Table 4). Protocadherin are a large family of cadherin-related molecules that are highly expressed in the brain and their expression appears to be developmentally regulated [32].

**Table 3:** Enriched GO terms in biological processes for SOX2 DEGs

GO CATEGORY	TOTAL GENES	CHANGED GENES	ENRICHMENT	LOG10(p)	FALSE DISCOVERY RATE
GO:0030935_sheet-forming_collagen	5	4	30.33	-5.64	0.00
GO:0042035_regulation_of_cytokine_biosynthetic_process	43	7	6.17	-3.93	0.04
GO:0005576_extracellular_region	561	30	2.03	-3.77	0.04
GO:0042107_cytokine_metabolic_process	48	7	5.53	-3.62	0.05
GO:0002376_immune_system_process	473	26	2.08	-3.51	0.05
GO:0042089_cytokine_biosynthetic_process	47	7	5.65	-3.68	0.05
GO:0044421_extracellular_region_part	392	24	2.32	-3.98	0.06
GO:0006952_defense_response	320	20	2.37	-3.51	0.06
GO:0005581_collagen	24	5	7.90	-3.46	0.07
GO:0042108_positive_regulation_of_cytokine_biosynthetic_process	29	5	6.54	-3.06	0.07
GO:0042226_interleukin-6_biosynthetic_process	8	3	14.22	-3.04	0.08
GO:0045408_regulation_of_interleukin-6_biosynthetic_process	8	3	14.22	-3.04	0.08
GO:0001525_angiogenesis	59	7	4.50	-3.06	0.08
GO:0005796_Golgi_lumen	7	3	16.25	-3.23	0.08
GO:0050926_regulation_of_positive_chemotaxis	7	3	16.25	-3.23	0.08
GO:0050927_positive_regulation_of_positive_chemotaxis	7	3	16.25	-3.23	0.08
GO:0032635_interleukin-6_production	17	4	8.92	-3.07	0.08
GO:0032675_regulation_of_interleukin-6_production	17	4	8.92	-3.07	0.08
GO:0042445_hormone_metabolic_process	57	7	4.66	-3.15	0.08
GO:0044420_extracellular_matrix_part	46	6	4.95	-2.91	0.08
GO:0032101_regulation_of_response_to_external_stimulus	62	7	4.28	-2.93	0.09
GO:0051093_negative_regulation_of_developmental_process	271	17	2.38	-3.08	0.09
GO:0050918_positive_chemotaxis	9	3	12.64	-2.87	0.09
GO:0009605_response_to_external_stimulus	415	22	2.01	-2.84	0.09

**Table 4:** Examples of SOX2 regulated genes and families

Gene Symbol	ratio *SOX2*_KO/MOCK	Description
**SOX family**		
SOX1	0.32	SRY (sex determining region Y)-box 1
SOX18	0.50	SRY (sex determining region Y)-box 18
*SOX2*	0.30	SRY (sex determining region Y)-box 2
		
**Brain-expressed**		
*BASP1*	3.52	brain abundant, membrane attached signal protein 1
*BEX2*	3.29	brain expressed X-linked 2
*BEX1*	4.81	brain expressed, X-linked 1
		
**G protein-coupled receptor**		
*GPR1*	4.22	G protein-coupled receptor 1
*GPR172B*	2.07	G protein-coupled receptor 172B
*GPR37*	0.49	G protein-coupled receptor 37
		
**Interleukins and their receptors**		
*IL1RL1*	2.48	interleukin 1 receptor-like 1
*IL23A*	2.60	interleukin 23, alpha subunit p19
*IL24*	5.73	interleukin 24
*IL32*	2.42	interleukin 32
*IL6*	10.25	interleukin 6 (interferon, beta 2)
*IL7R*	2.32	interleukin 7 receptor
*IL8*	2.22	interleukin 8
		
**Solute carrier family**		
*SLC14A1*	4.81	solute carrier family 14 (urea transporter), member 1 (Kidd blood group)
*SLC2A3*	2.29	solute carrier family 2 (facilitated glucose transporter), member 3
*SLC22A1*	3.80	solute carrier family 22 (organic cation transporter), member 1
*SLC3A2*	2.07	solute carrier family 3 (activators of dibasic and neutral amino acid transport), member 2
*SLC30A1*	2.62	solute carrier family 30 (zinc transporter), member 1
*SLC7A1*	2.25	solute carrier family 7 (cationic amino acid transporter, y+ system), member 1
		
**Protocadherin family**		
*PCDH10*	0.45	protocadherin 10
*PCDH9*	0.32	protocadherin 9
*PCDHA4*	2.20	protocadherin alpha 4
*PCDHB11*	0.38	protocadherin beta 11
*PCDHGA3*	0.49	protocadherin gamma subfamily A, 3
*PCDHGC3*	0.43	protocadherin gamma subfamily C, 3

The expression of many interesting gene families were up regulated after *SOX2 *knockdown. For example, we found that brain expressed genes *BASP1 *(brain abundant, membrane attached signal protein 1), *BEX1 *(brain expressed X-linked 1) and *BEX2 *(brain expressed X-linked 2) were up regulated after *SOX2 *knockdown (Table 4). We also found that knocking down *SOX2 *also increase the expression of many solute carrier family proteins including *SLC2A3, SLC3A2, SLC7A1, SLC14A1, SLC22A1 and SLC30A1*. These solute carrier proteins transport many important solutes such as urea, glucose, organic cations, dibasic and neutral amino acids, zinc, and cationic amino acids (Table 4).

The gene regulated by *SOX2 *could be directly regulated or indirectly regulated. By integrating the array data with the ChIP-seq data, the directly targeted genes of *SOX2 *can be inferred. We found 88 *SOX2*-regulated genes whose promoters were bound by 127 *SOX2 *binding regions (Figure. 3D and Additional File 1). Interesting genes include *BCL2 *(B-cell CLL/lymphoma 2) and *BCL2 *interacting protein 3 (BNIP3), four brain and neuron expressed genes *BASP1 *(brain abundant, membrane attached signal protein 1), *NEDD4 *(Neural precursor cell expressed, developmentally down-regulated 4), *NRG1 *(Neuregulin 1) and *NEGR1 *(Neuronal growth regulator 1), two interleukins *IL6 *and *IL8*, two protocadherins *PCDH9 *and *PCDH10*, *RUNX1 *(Runt-related transcription factor 1, acute myeloid leukemia 1 oncogene), and three solute carrier proteins *SLC3A2, SLC7A1 *and *SLC30A1 *(Additional File 11). The VENN diagram also showed that 11 genes were common *SOX2 *targets for GBM and ES cells and changed in expression after *SOX2 *knockdown (Figure. 3D and Table 5). They include *EBF3 *(early B-cell factor 3), *BASP1 *(brain abundant, membrane attached signal protein 1), *SLC30A1 *(solute carrier family 30 (zinc transporter), member 1), and *SLC3A2 *(solute carrier family 3, member 2). We speculate that these genes may be involved in GBM stem cells. However, further experimentations are necessary to understand the role of these genes in glioma stem cells.

**Table 5:** Common SOX2 targets of human GBM and ES cells that changed expression after SOX2 knockdown

Gene Symbol	Mock/*SOX2*_KO ratios	Description
*ALCAM*	2.02	activated leukocyte cell adhesion molecule
*BASP1*	0.28	brain abundant, membrane attached signal protein 1
*BCAT1*	0.38	branched chain aminotransferase 1, cytosolic
*COL12A1*	2.17	collagen, type XII, alpha 1
*CTH*	0.37	cystathionase (cystathionine gamma-lyase)
*EBF3*	2.03	early B-cell factor 3
*KLF5*	0.49	Kruppel-like factor 5 (intestinal)
*ONECUT1*	0.48	one cut domain, family member 1
*PTHLH*	0.34	parathyroid hormone-like hormone
*SLC30A1*	0.38	solute carrier family 30 (zinc transporter), member 1
*SLC3A2*	0.48	solute carrier family 3 (activators of dibasic and neutral amino acid transport), member 2

### *SOX2 *and miR145 form a double-negative feedback loop in GBM cells

We also analyzed the effect of *SOX2 *on miRNAs using next generation sequencing (Illumina). MicroRNA sequencing is an efficient way to identify known and novel microRNAs that are differentially expressed [33]. After miRNA sequencing and data analysis, we found 105 precursor microRNAs (corresponding to 95 mature miRNAs) that were changed > 2 fold between *SOX2 *knockdown and *SOX2*-MOCK transfected LN229 cells (Table 6 and Additional File 12). Six microRNAs including miR-145, -143, -145*, -143*, -253-5p and miR-452, were up regulated after *SOX2 *knockdown and the rest were down regulated when *SOX2 *was knocked down. We picked 6 miRNAs to confirm the next generation data using RT-PCR. We confirmed that miR-143 and miR-145 were up regulated after *SOX2 *knockdown and that miR-146a, -25, -20b and miR-9-1 were down regulated after *SOX2 *knockdown (Figure. 3B).

**Table 6:** SOX2 regulated miRNAs identified by next generation sequencing

precursor miRNA ID	mature miRNA ID	MOCK/*SOX2 *KO	P value
hsa-mir-106a	hsa-miR-106a	17.89	1.94E-68
hsa-mir-106a	hsa-miR-106a*	20.50	2.41E-06
hsa-mir-106b	hsa-miR-106b*	2.68	2.30E-05
hsa-mir-106b	hsa-miR-106b	2.55	9.03E-05
hsa-mir-10b	hsa-miR-10b	2.70	1.09E-05
hsa-mir-125b-1	hsa-miR-125b	2.80	2.79E-06
hsa-mir-125b-1	hsa-miR-125b-1*	2.62	6.78E-05
hsa-mir-125b-2	hsa-miR-125b	2.80	2.75E-06
hsa-mir-1268	hsa-miR-1268	2.85	5.28E-06
hsa-mir-1271	hsa-miR-1271	3.44	0.000102818
hsa-mir-1287	hsa-miR-1287	6.63	6.21E-17
hsa-mir-1301	hsa-miR-1301	3.60	5.03E-06
hsa-mir-1305	hsa-miR-1305	3.28	2.17E-05
hsa-mir-1307	hsa-miR-1307	4.36	6.38E-16
hsa-mir-130b	hsa-miR-130b*	3.97	2.19E-11
hsa-mir-135b	hsa-miR-135b*	5.26	8.40E-19
hsa-mir-135b	hsa-miR-135b	5.09	2.86E-17
hsa-mir-140	hsa-miR-140-3p	8.69	2.82E-41
hsa-mir-140	hsa-miR-140-5p	7.93	4.29E-37
hsa-mir-143	hsa-miR-143	0.02	3.04E-182
hsa-mir-143	hsa-miR-143*	0.05	2.41E-72
hsa-mir-145	hsa-miR-145	0.02	1.99E-132
hsa-mir-145	hsa-miR-145*	0.03	4.74E-113
hsa-mir-146a	hsa-miR-146a	17.38	1.05E-73
hsa-mir-146a	hsa-miR-146a*	11.40	7.59E-07
hsa-mir-149	hsa-miR-149	4.27	3.27E-14
hsa-mir-15b	hsa-miR-15b*	3.18	3.05E-05
hsa-mir-17	hsa-miR-17	3.28	2.85E-09
hsa-mir-187	hsa-miR-187	9.55	1.40E-09
hsa-mir-188	hsa-miR-188-5p	4.83	2.82E-08
hsa-mir-18a	hsa-miR-18a	3.46	1.76E-09
hsa-mir-18b	hsa-miR-18b	21.77	1.55E-47
hsa-mir-190	hsa-miR-190	2.78	2.00E-05
hsa-mir-196a-1	hsa-miR-196a	2.58	5.85E-05
hsa-mir-196a-2	hsa-miR-196a	2.55	9.28E-05
hsa-mir-1977	hsa-miR-1977	0.32	4.88E-07
hsa-mir-200c	hsa-miR-200c	2.63	5.38E-05
hsa-mir-204	hsa-miR-204	38.20	4.44E-83
hsa-mir-20a	hsa-miR-20a	3.03	1.10E-07
hsa-mir-20b	hsa-miR-20b	24.84	1.62E-88
hsa-mir-20b	hsa-miR-20b*	16.86	4.09E-28
hsa-mir-217	hsa-miR-217	6.54	1.10E-10
hsa-mir-224	hsa-miR-224*	0.13	4.22E-18
hsa-mir-2276	hsa-miR-2276	11.25	2.06E-15
hsa-mir-25	hsa-miR-25	2.92	5.28E-07
hsa-mir-28	hsa-miR-28-3p	2.70	1.25E-05
hsa-mir-30b	hsa-miR-30b	4.91	1.20E-19
hsa-mir-30d	hsa-miR-30d	4.80	2.04E-19
hsa-mir-30d	hsa-miR-30d*	4.79	8.38E-12
hsa-mir-320a	hsa-miR-320a	2.54	0.000106251
hsa-mir-330	hsa-miR-330-5p	3.90	5.23E-06
hsa-mir-338	hsa-miR-338-5p	42.70	2.78E-75
hsa-mir-338	hsa-miR-338-3p	32.33	6.56E-64
hsa-mir-339	hsa-miR-339-5p	2.75	4.52E-05
hsa-mir-340	hsa-miR-340*	4.53	5.95E-10
hsa-mir-361	hsa-miR-361-3p	2.66	6.29E-05
hsa-mir-362	hsa-miR-362-5p	4.32	8.19E-13
hsa-mir-363	hsa-miR-363	21.08	2.70E-82
hsa-mir-423	hsa-miR-423-3p	3.19	1.05E-08
hsa-mir-452	hsa-miR-452	0.20	4.36E-59
hsa-mir-454	hsa-miR-454*	7.09	8.55E-13
hsa-mir-454	hsa-miR-454	2.79	7.68E-05
hsa-mir-484	hsa-miR-484	3.32	1.74E-08
hsa-mir-500	hsa-miR-500*	4.18	2.46E-14
hsa-mir-501	hsa-miR-501-3p	6.15	2.77E-19
hsa-mir-501	hsa-miR-501-5p	5.17	6.25E-11
hsa-mir-502	hsa-miR-502-3p	4.19	9.80E-11
hsa-mir-514-1	hsa-miR-514	9.25	0.000108265
hsa-mir-514-2	hsa-miR-514	9.25	0.000108265
hsa-mir-514-3	hsa-miR-514	9.25	0.000108265
hsa-mir-532	hsa-miR-532-5p	4.74	2.71E-18
hsa-mir-532	hsa-miR-532-3p	3.72	1.47E-07
hsa-mir-548d-1	hsa-miR-548d-5p	3.52	2.17E-08
hsa-mir-548d-2	hsa-miR-548d-5p	3.45	3.03E-08
hsa-mir-548n	hsa-miR-548n	3.84	2.66E-08
hsa-mir-573	hsa-miR-573	5.67	6.41E-06
hsa-mir-574	hsa-miR-574-5p	4.68	8.73E-18
hsa-mir-577	hsa-miR-577	14.88	6.79E-60
hsa-mir-584	hsa-miR-584	9.12	1.41E-42
hsa-mir-585	hsa-miR-585	46.00	2.35E-14
hsa-mir-589	hsa-miR-589*	3.76	3.23E-08
hsa-mir-589	hsa-miR-589	2.83	2.95E-06
hsa-mir-590	hsa-miR-590-3p	3.15	6.09E-06
hsa-mir-592	hsa-miR-592	24.45	1.71E-52
hsa-mir-615	hsa-miR-615-3p	3.79	5.88E-09
hsa-mir-616	hsa-miR-616	3.26	6.21E-05
hsa-mir-626	hsa-miR-626	0.12	2.40E-10
hsa-mir-652	hsa-miR-652	3.99	1.57E-12
hsa-mir-660	hsa-miR-660	4.27	1.17E-10
hsa-mir-671	hsa-miR-671-5p	2.73	8.59E-06
hsa-mir-671	hsa-miR-671-3p	2.83	5.90E-05
hsa-mir-760	hsa-miR-760	10.74	1.39E-27
hsa-mir-873	hsa-miR-873	11.50	1.21E-14
hsa-mir-877	hsa-miR-877	4.22	2.51E-13
hsa-mir-889	hsa-miR-889	0.16	3.89E-05
hsa-mir-9-1	hsa-miR-9	10.74	2.37E-50
hsa-mir-9-1	hsa-miR-9*	11.87	3.43E-35
hsa-mir-9-2	hsa-miR-9	10.74	2.39E-50
hsa-mir-9-2	hsa-miR-9*	11.87	3.43E-35
hsa-mir-9-3	hsa-miR-9	10.74	2.37E-50
hsa-mir-9-3	hsa-miR-9*	11.06	2.66E-35
hsa-mir-92a-1	hsa-miR-92a	3.38	5.65E-10
hsa-mir-92a-2	hsa-miR-92a	3.35	8.85E-10
hsa-mir-93	hsa-miR-93	3.46	1.43E-10
hsa-mir-942	hsa-miR-942	3.43	3.82E-07

Xu *et al*. showed that miR-145 targets *SOX2 *and down regulates its expression in human embryonic stem cells [34]. To see whether the same is true in GBM cells, we transfected LN229 GBM cell with miR-145 mimics and we found that miR-145 also decreased *SOX2 *expression in GBM cells (Figure. 3C). As knocking down *SOX2 *up regulates miR145 in the RT-PCR and next-generation sequencing data (Figure. 3B and Table 6), this suggests that *SOX2 *itself down regulates miR-145. Taken together, *SOX2 *down regulates miR-145 and miR145 also down regulates *SOX2*, suggesting that *SOX2 *and miR145 form a double-negative feedback loop in GBM cells. We also checked to see whether there are *SOX2 *binding regions in the proximity of miR145 genomic locus. We found that there are no SOX2 binding regions with significant P values (P < 0.01) in the close proximity of the miR145 locus. The closest one is about 23 kb from the miR145 genomic locus. This may suggest that the *SOX2 *feedback regulation of miR145 is indirect, not resulting from direct binding of the *SOX2 *to the miR145 genomic region.

## Discussion

We applied ChIP-seq technology to identify global *SOX2 *binding regions in GBM cells. To our best knowledge, this is the first global analysis of *SOX2*'s binding regions in cancer cells. *SOX2 *encodes a member of the SRY-related HMG-box (*SOX*) family of transcription factors. We investigated *SOX2*'s global binding targets by ChIP-Seq analysis, and found that *SOX2 *binding regions in GBM cells are enriched for AT nucleotides with a consensus sequence wwTGnwTw [w = A or T; its reverse and complement strand wAwnCAww]. The mouse *sox2 *consensus motif in the mouse ES cells found by Chen *et al*. has the sequence 5'-CATTGTT-3' [26]. The similarity lies in that both consensus sequences are AT rich sequences with a core TG di-nucleotide flanked by AT rich sequences. The difference may due to the fact that they derived from different types of cells (ES vs. glioma) and species (human vs. mouse).

The AT rich sequence we identified for *SOX2 *consensus is consistent with previous *in vitro *studies showing that the HMG domain of SOX proteins binds to the minor groove of DNA through AT rich sequences with a heptamer motif WWCAAAG (W = A or T) [35,36]. Therefore we have identified AT rich *SOX2 *specific binding sequences. Before the development of ChIP-chip or ChIP-Seq technologies, Mertin *et al*. determined the DNA-binding properties of *SOX9 *using random oligonucleotide selection assay [37] and they identified a core sequence of an AT rich sequence AACAAT or wwCAAw (w, A or T) for *SOX9 *binding. The HMG domain in SOX family proteins forms an L-shaped module composed of three helices that binds to DNA in the minor groove. SOX proteins are categorized into Groups A-G based on their sequence homology [38]. *SOX2 *belongs to Group A (also named SRY) and *SOX9 *belongs to group E [38]. The amino acid sequence identity of the HMG domain within the same group is high at >90%, however, the amino acid sequence identity between distant groups decreases to ~60% [38]. A sequence alignment revealed that *SOX2 *and *SOX9 *only have about 61% amino acid sequence identity in the HMG domain. The sequence variations may explain the similar AT rich properties yet different consensus in their binding regions for *SOX2 *and *SOX9*. Additional functional binding assays including mutagenesis and footprinting analysis will be needed to confirm the binding activities and specificities. Further experimentation is therefore warranted.

It was a surprise to find that about one quarter of genes regulated by *SOX2 *encompass important GO categories: 196 out of 792 genes (about 25%) were found to have signal transducer activity (GO:0004871), 101 of 410 belong to transmembrane receptor genes (about 25%) (GO: 0004888), and 92 of 365 are kinase genes (about 25%) (GO:0016301). Signal transducer, receptor and kinase genes are important genes that play an essential role in cellular functions and therefore it is not surprising that *SOX2 *is an essential gene that plays important roles in development and in carcinogenesis.

We found that *BEX1 *(brain expressed X-linked 1) and *BEX2 *(brain expressed X-linked 2) were up regulated after *SOX2 *knockdown (Table 4). We have previously shown that *BEX1 *and *BEX2 *are silenced in GBM tumor specimens and exhibited extensive promoter hypermethylation [39]. We demonstrated by *in vitro *and in a xenograft mouse model that *BEX1 *or *BEX2 *possess tumor suppressor activity [39]. Our data suggested that *SOX2 *might down regulate *BEX1 *and *BEX2 *expression, reducing their tumor suppressor activities and thus promoting carcinogenesis. However, we did not find *SOX2 *binding regions in the *BEX1 *and *BEX2 *gene loci, suggesting the down regulation was properly an indirect effect of *SOX2 *knockdown.

We found that *SOX2 *also regulates the expression of SOX family protein *SOX1 *and *SOX18 *(Table 4). *SOX1 *plays roles in neural determination and differentiation [40] and is a neural stem cell marker [41]. Bylund *et al*. showed that *sox1*, sox2 and *sox3 *are the transcription factors that keep neural cells undifferentiated by counteracting the activity of proneural proteins [42]. However, the role of *SOX1 *in GBM has not yet been studied. *SOX18 *plays important roles in blood vasculature formation [43]. Young *et al*. assessed the effects of disrupted *SOX18 *function on MCF-7 human breast cancer and human umbilical vein endothelial cell (HUVEC) proliferation by measuring BrdU incorporation and by MTT assay, cell migration using Boyden chamber assay, and capillary tube formation in vitro [44]. They showed that over expression of wild-type *SOX18 *promoted capillary tube formation of HUVECs in vitro, whereas expression of dominant-negative *SOX18 *impaired tube formation of HUVECs [44]. Therefore, *SOX18 *is a potential target for antiangiogenic therapy of human cancers. The role of *SOX18 *in GBM has not been studied. Taking together, *SOX2 *could act through *SOX1 *and *SOX18*, and thus play roles in both maintaining stem cell properties of glioma cells and forming tumor vasculature in gliomas, which are two major obstacles preventing us from treating these tumors effectively.

By microRNA sequencing we determined that levels of 105 precursor microRNAs (corresponding to 95 mature miRNAs) are altered in response to *SOX2 *knockdown (Table 6 and Additional File 12). We showed that *SOX2 *could down regulate the expression of miR-143 and miR-145. miR-145 was shown to be down regulated in several cancers such as colon cancers [45] and prostate cancers [46], and miR-143 was shown to be down regulated in colon cancers [47] and bladder cancers [48]. The relationship of miR-143 and miR-145 and GBM has not been studied and is worth future investigation.

**Figure 4:**
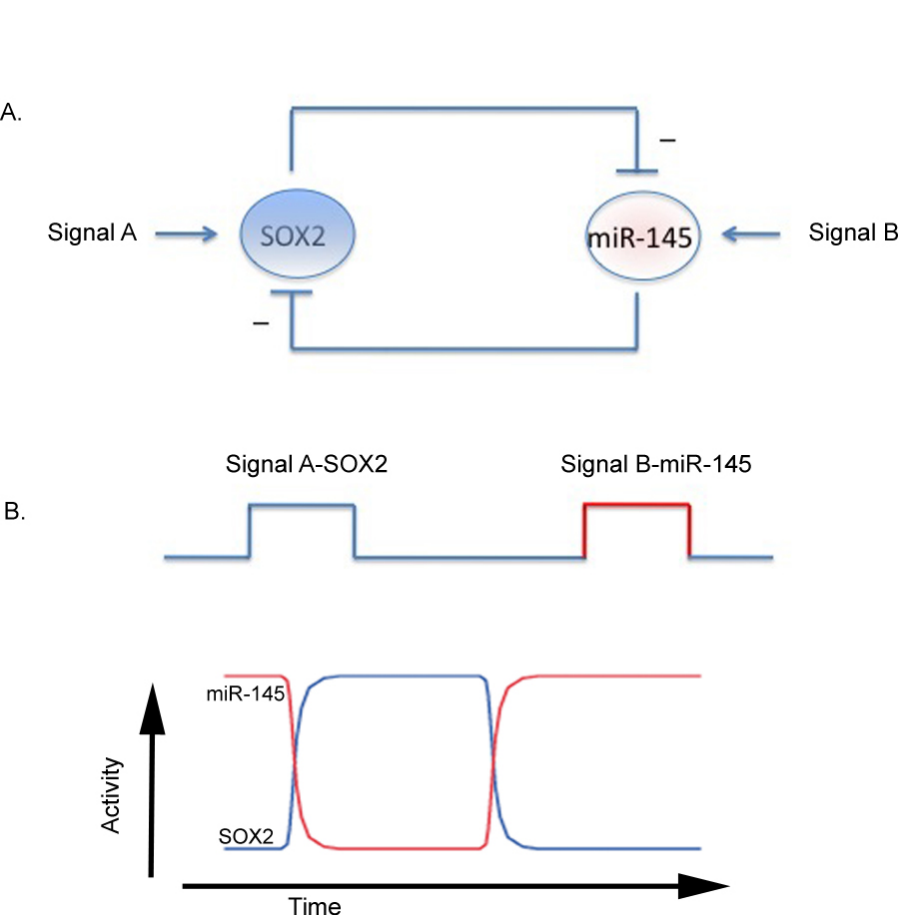
**The double negative feedback loop of *SOX2 *and miR-145**. (A) An illustrative drawing showing the double negative feedback loop of *SOX2 *and microRNA 145. Negative signs and upside down T line drawing indicate inhibitive action. Both *SOX2 *and miR-145 can receive separate input signals (signal A or signal B). (B) Theoretical bistable output of this double negative feedback loop. Depending on the strength of the input signal A or B, the system could toggle between two stable states: the signal A-*SOX2 *or the signal B-miR-145 states.

We further demonstrated that *SOX2 *and miR-145 form a double-negative regulation loop in GBM cells (Figure. 4A). Double-negative feedback loop involving microRNAs and their targets have been observed previously [49,50]. A double-negative feedback mechanism has been proposed as a mechanism to form bistability in cellular states. Johnston *et al*. demonstrated that the stability and irreversibility of the terminal differentiated state of neuronal cells is ensured by a double-negative feedback loop between two microRNAs *lsy-6 *and *mir-273 *and their transcription factor targets in the nematode *Caenorhabditis elegans *[50]. A positive-feedback loop or double-negative feedback loops can convert graded inputs into switch-like, irreversible responses [51]. Such a system will be "bistable" as the system exists almost exclusively in one of two possible states. Bistability has been shown in several signal transduction and transcriptional regulatory events such as the p42 mitogen-activated protein kinase and c-Jun amino-terminal kinase pathways in *Xenopus oocytes *[51]. For a bistable system with two components A and B, the system will toggle between two stable states: one with A on and B off and one with B on and A off. For example, for the *SOX2 *and miR-145 bistable system, the system can be on *SOX2 *on, miR-145 off state or *SOX2 *off, miR-145 on state (Figure. 4B). Further experimentation will be necessary to analyze in detail the two cellular states for the *SOX2*-miR145 double-negative feedback loop in GBM cells.

## Conclusion

We have comprehensively characterized the *SOX2 *response program by integrated analysis using several advanced technologies including ChIP-seq, microarrays and microRNA sequencing. The datasets of ChIP-seq, microarrays and microRNA sequencing of *SOX2 *response program, which, to our best knowledge, are the first datasets of *SOX2 *in cancers, will be useful resources for the research community. Furthermore, the insights we gained from our integrated analysis further our understanding of the roles of *SOX2 *in carcinogenesis.

## Methods

### Cell culture and functional assays

LN229 cells were obtained from the American Type Culture Collection (Manassas, VA) and maintained in DMEM with 10% fetal bovine serum. Cell proliferation was analyzed using the MTT assay kits (Millipore, Billerica, MA) according to the manufacturer's protocol. For Soft agar colony formation assay, cells were trypsinized and counted. 10,000 cells were seeded in six-well plates. After 2 weeks of growth, colonies with a diameter greater than 4 mm were counted. Experiments were performed in quadruplicates [39].

### Chromatin immunoprecipition (ChIP) - Sequencing

About 3 × 10^6 ^LN229 cells were used for chromatin immunoprecipition (ChIP) assay, carried out according to the manufacturer's instructions (Millipore, EZ-Magna ChIP™ A). Antibodies used for ChIP included *SOX2 *(ab59776, Abcam Inc.) and IgG (sc-2027, Santa Cruz Biotehnology Inc.). For ChIP, *SOX2 *antibody was tested for its specificity and specific band was found (Figure 1A). ChIP assay was performed using the ChIP kit (Upstate Biotechnology, Lake Placid, NY) according to the manufacturer's protocol. Briefly, cells were cross-linked by adding fresh formaldehyde to cell culture medium to a final concentration of 1%. Fixation was monitored at 37°C for 10 min. The fixed cells were re-suspended in the lysis buffer. Nuclei were collected by centrifugation at 2000 × ***g***, and resuspended in the nuclei lysis buffer. Samples were sonicated on ice to the length of 200-500 base pairs. 5 μg antibody and 50 μl Dynal protein G beads were incubated for 2 hours at 4°C. Sonicated chromatin were incubated with the protein G- antibody complex overnight at 4°C. Precipitated immunocomplex was treated with proteinase K for 2 hours at 65°C, and DNA was purified Qiagen Qiaquick PCR purification kit. ChIP DNA end repairing, adaptor ligation, and amplification were performed as described earlier [23]. Fragments of about 200 bp (without linkers) were isolated from agarose gel and used for sequencing using the Illumina 2 G genetic analyzer. Illumina data analysis pipeline was performed as described [23]. For this manuscript, the same genome build hg18 and its associated annotations were used for all analysis. Sequence reads that map to multiple sites in the human genome were removed. To identify *SOX2 *binding peaks, we used SISSRs (Site Identification from Short Sequence Reads) (http://www.rajajothi.com/sissrs/) [25] with default parameters with E-value is set to 10, P value set to 0.001. *SOX2 *ChIP-seq as positive and IgG control ChIP-seq data as negative input.

To calculate the distance to the TSS start site, annotations from the UCSC (hg18) were used. We also took into consideration the direction of the strand when we calculated the distance to TSS. As the *SOX2 *binding regions is always recorded on the positive strands, for genes mapped to the positive strand, the distance is the end position of the *SOX2 *binding region minus the TSS start position; for genes mapped to the negative strand, the distance is the TSS start position minus the *SOX2 *binding region start position.

### Validation of ChIP-seq datasets by ChIP-qPCR

We selected a list of binding peaks for validation using quantitative real-time PCR. The primers were listed in the Additional File 13. Three replicates were run. Real-time PCR was performed using the SYBR^® ^Green (Invitrogen) dye detection method on ABI PRISM 7900 HT Sequence Detection System under default conditions: 95°C for 10 min, and 35 cycles of 95°C for 15 s and 55°C for 1 min. Comparative C_t _method was used for quantification of the transcripts.

### Gene Ontology Analysis

High-Throughput GoMiner [52] was used to find statistically over represented Gene Ontology (GO) terms. GO terms of all evidence levels and categories were used for the analysis. The algorithm used by the High-throughput GoMiner [52], which is the one-sided Fisher exact p value corrected for multiple comparisons, was used to calculate the FDR (false discovery rate). To identify over represented GO terms in the 3162 unique *SOX2 *targets in GBM cells versus the 817 unique *SOX2 *targets in human ES cells, we GSEA (http://www.broadinstitute.org/gsea/index.jsp). The parameters used were: 1000 permutations using the C5 gene sets (GO gene sets), the diff_of_classes algorithm as metric for ranking genes, weighted enrichment statistic, minimum gene set size of 3. Other parameters were set as default.

### Motif scanning and identification

To identify novel motifs, *SOX2 *binding regions identified by ChIP-seq were extended to 100 bp 5' and 3' and the sequences were retrieved in FASTA format. The sequences were first subjected to the RepeatMask program (http://www.repeatmasker.org/) to mask all human repeats. We used the MotifSampler to find over-expressed motifs in the *SOX2 *binding regions with the default parameters. The over represented sequences were used as input for the Weblog program (http://weblogo.berkeley.edu/) [53] to display the consensus sequence graphically. For a systematic search for all potential transcription binding sites, we used the Motifscanner software (http://bioinformatics.psb.ugent.be/webtools/plantcare/html/Motif_Sampler.html). Human upstream sequences from EPD (The Eukaryotic Promoter Database) (epd_homo_sapiens_499_chromgenes_non_split_3.bg) were downloaded from the motif scanner web site and used as the background model. The human subset of the Transfac professional 7.0 PWM matrices was used. Matched TF matrices with likelihood ratios (LR) of 500 or higher were tabulated and their frequencies calculated.

### Small interfering RNA transfection

*SOX2 *SiRNAs (Ambion Inc) were used for transient knockdown of *SOX2*. The SiRNA sequences are: s13295, sense sequence AGUGGAAACUUUUGUCGGATT and antisense sequence, UCCGACAAAAGUUUCCACUCG; S13296, sense sequence ACCAGCGCAUGGACAGUUATT and anti-sense sequence UAACUGUCCAUGCGCUGGUTC. LN229 cells were seeded into six well plates, cultured overnight and transfected with *SOX2 *SiRNAs at a final concentration of 100 nM using TransIT-OT1 (Mirus Bio LLC, Madison WI) according to the manufacturer's instructions. At 72 hours after tansfection, cells were harvested for western blot analysis and for microarray analysis.

### Microarray analysis

The Applied Biosystems' microarray platform was used using the standard array hybridization protocol as we described previously [39]. The ABI arrays contain 31,700 60-mer oligonucleotide probes representing 29,098 individual human genes. Two biological replicates were performed including cell transfection and microarray analysis. GeneSpring program were used to analyze the array data. The raw signal intensities individual probes were combined (averaged) based on Celera's Gene ID (ABI's annotation table), and then imported into the GeneSpring program, and data from individual chip were normalized per chip with 75% percentile and normalized per gene by median. To filter out those lowly expressed genes across all chips, only arrays data with a signal to noise (S/N) ratio of > 3 in one of the arms (*SOX2 *Knockdown or mock knockdown) were used for analysis. Two biological replicates were performed. The normalized data for the replicates were averaged for each gene. To identify differentially expressed genes, a two fold cutoff value was used.

### Integrative analysis

The human genome build hg18 and its associated annotations were used for all analysis. The *SOX2 *binding regions identified by ChIP-seq (Table S2) was annotated with the genome annotation of hg18 for their associated genes using the nearest gene within 50 kb of the TSS (transcription start site) or TES (transcription end site). As the published human ChIP-Chip data in human embryonic stem cells [27] was annotated in 2005 with human genome hg17 when the paper was published, the annotation was outdated. We first converted the *SOX2 *binding regions coordinates (Table S3 of the Boyer's paper) into the hg18 coordinates using the tool liftover (http://genome.ucsc.edu/cgi-bin/hgLiftOver). We re-annotated the human ES *SOX2 *binding regions with hg18 annotations using the chromosomal coordinates with the same criteria as we used for the GBM *SOX2 *ChIP-seq data. There were 1,075 human ES *SOX2 *binding regions that could be annotated with nearby genes. To identify overlapping genes between *SOX2 *ChIP-seq and the microarray data, we used the gene symbols from the HUGO gene nomenclature committee (http://www.genenames.org/) to compare the two lists. To compare the human ChIP-seq data with the *sox2 *targets that were identified in mouse ES cells [26], we used the homologene table for human and mouse from NCBI (http://www.ncbi.nlm.nih.gov/homologene) to identify the human homologues for the mouse *sox2 *targets and then used the human homologues to compare with the human *SOX2 *ChIP-seq data.

### RNA isolation and Small RNA sequencing

RNA was isolated from cells using mirVana™ miRNA Isolation Kit (Ambion, Austin, TX) according to the manufacturer's instructions. Sequencing library preparation was carried out according to Illumina mirna sample preparation protocol. Small RNA samples were sequenced using GA2 sequencer (Illumina)

For data analyses, Solexa adapters were first trimmed from raw sequences using custom Perl scripts, and the trimmed sequences were then aligned to known human miRNAs precursors (miRBase release 14) using miRExpress[54]. The -t parameter (alignment identity between query and reference sequences) for miRExpress was set to be 0.9. The expression abundance of corresponding miRNAs were counted by miRExpress and normalized by the counts of trimmed sequences in the library and used for further analysis.

Differentially expressed miRNAs were identified by statistics analysis using edgeR package from bioconductor[55]. The top differentially expressed ones were clustered by MeV[56,57].

### Validation of miRNAs expression by RT-PCR

We selected a list of miRNAs for validation using quantitative real-time PCR. The primers were available upon request. Three replicates were run. Real-time PCR was performed using the SYBR^® ^Green (Invitrogen) dye detection method on ABI PRISM 7900 HT Sequence Detection System under default conditions: 95°C for 30 s, and 40 cycles of 95°C for 15 s and 60°C for 20 s. Comparative Ct method was used for quantification of the transcripts.

### Transfection of microRNA

microRNA-145 precursor mimics was obtained from Ribobio company (Guanzhou China). A scrambled precursor with no homology to the human genome was used as controls. LN229 cells were transfected with the precursor mimics by lipofectamine 2000 (Invitrogen).

### Data access

The array data for *SOX2 *knockdown and control data from this study have been submitted to Gene Expression Omnibus (GEO) under accession No.GSE23839. The *SOX2 *ChIP-seq data from this study have been submitted to Gene Expression Omnibus (GEO) under accession No. GSE23795.

## Abbreviations

ChIP-seq: Chromatin immunoprecipitation-Sequencing; SRY: sex determining region Y; ShRNA: small hairpin RNA.

## Authors' contributions

BL and XF designed this study. XF, JY, LL performed the experiments. WY performed data analysis. SZ, LH, DRG, GF help to design the study and wrote the paper. All authors read and approved the final manuscript.

## Supplementary Material

Additional file 1 *SOX2 *binding regions in GBM cells identified by ChIP-seq. Click here for file

Additional file 2 **Common genes from the two *SOX2 *Chip_seq datasets from mouse ES cells**. Click here for file

Additional file 3 **Over represented GO terms in 3162 unique SOX2 targets in GBM cells**. Click here for file

Additional file 4 **Over represented GO terms in 817 unique SOX2 targets in human ES cells**. Click here for file

Additional file 5 **SOX2 targets that are related to regulation of cell differentiation defined by GO term GO:0045595 (REGULATION OF CELL DIFFERENTIATION)**. Click here for file

Additional file 6 **SOX2 targets that are related to stemness defined by 378 genes overexpressed in human embryonic stem cells according to 5 or more out of 20 profiling studies in the Table S1 of Ben-Porath *et al.*'s paper **[28]. Click here for file

Additional file 7 **The *SOX2 *consensus matrix**. Click here for file

Additional file 8 **The frequency of the matches to the *SOX2 *matrix**. Click here for file

Additional file 9 **Frequencies of known TFs binding sites co-occurred in the *SOX2 *binding regions**. Click here for file

Additional file 10 **Differentially expressed genes between *SOX2*-Knockdown cells and control cells**. Click here for file

Additional file 11 ***SOX2 *binding and changed in gene expression**. Click here for file

Additional file 12 ***SOX2 *regulated miRNAs identified by Illumina's next generation sequencing**. Click here for file

Additional file 13 **Primers for Q-RT-PCR for confirmation of ChIP-Seq data**. Click here for file
